# Recent intensification of Amazon flooding extremes driven by strengthened Walker circulation

**DOI:** 10.1126/sciadv.aat8785

**Published:** 2018-09-19

**Authors:** Jonathan Barichivich, Emanuel Gloor, Philippe Peylin, Roel J. W. Brienen, Jochen Schöngart, Jhan Carlo Espinoza, Kanhu C. Pattnayak

**Affiliations:** 1 Instituto de Conservación, Biodiversidad y Territorio, Universidad Austral de Chile, Valdivia, Chile.; 2 Center for Climate and Resilience Research, Santiago, Chile.; 3 School of Geography, University of Leeds, Leeds, UK.; 4 Laboratoire des Sciences du Climat et de l‘Environnement, CEA CNRS UVSQ, Gif-sur-Yvette 91190, France.; 5 Instituto Nacional de Pesquisas da Amazônia, Coordenação de Dinâmica Ambiental, Manaus, Brazil.; 6 Instituto Geofísico del Perú (IGP), Subdirección de Ciencias de la Atmósfera e Hidrósfera (SCAH), Lima, Perú.

## Abstract

The Amazon basin is the largest watershed on Earth. Although the variability of the Amazon hydrological cycle has been increasing since the late 1990s, its underlying causes have remained elusive. We use water levels in the Amazon River to quantify changes in extreme events and then analyze their cause. Despite continuing research emphasis on droughts, the largest change over recent decades is a marked increase in very severe floods. Increased flooding is linked to a strengthening of the Walker circulation, resulting from strong tropical Atlantic warming and tropical Pacific cooling. Atlantic warming due to combined anthropogenic and natural factors has contributed to enhance the change in atmospheric circulation. Whether this anomalous increase in flooding will last depends on the evolution of the tropical inter-ocean temperature difference.

## Teaser

A recent increase in extreme flooding in Amazonia is linked to intensification of the atmospheric east-west Walker circulation.

## INTRODUCTION

The Amazon basin and its extensive tropical rainforests sustain one of the major centers of deep atmospheric convection and heavy rainfall on the planet. Changes in the water cycle in this region both affect and respond to the global Hadley and Walker circulations and also have major impacts on the global carbon cycle through variations in the carbon balance of the rainforests (*1*). There is mounting evidence that the hydrological cycle of the basin has intensified since the late 1990s, with more frequent hydrological extremes causing major human suffering and disturbance to the rainforest ecosystems (*1*–*4*). In particular, strong and extensive droughts in central and southern Amazonia in 2005, 2010, and 2015 have raised concerns that drying trends expected as a consequence of deforestation (*5*, *6*) or projections by some climate models under scenarios of climate change may have begun to be realized (*4*, *7*, *8*). However, despite recent research emphasis on droughts, an even more prominent feature of change in Amazon hydrology is the recent occurrence of severe floods (*2*–*4*). Severe floods have become a recurrent disaster in northwestern Amazonia and along the Amazon River and some of its tributaries from 2009 onward (*9*–*11*). While a number of studies have documented concurrent changes in the tropical climate system, the mechanisms and ultimate cause of the intensification of the hydrological cycle in the basin and, particularly, the increase in the occurrence of very severe floods remain unclear (*3*, *4*). Here, we first document and quantify long-term changes in hydrological extremes in Amazonia and then identify their main driving mechanisms and discuss the implications for Amazonian climate over the next decades.

## RESULTS

**Fig. 1:**
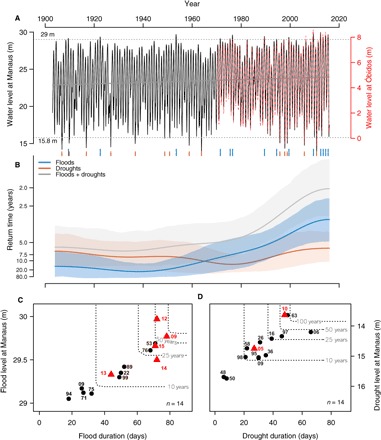
Frequency, severity, and duration of historical extreme floods and droughts in Amazonia. (**A**) Daily water level of Negro River at Manaus (black; 1903–2015) and Amazon River at Óbidos (red; 1968–2015). The horizontal dotted lines denote the thresholds used to define drought (15.8 m) and flood (29.0 m) events from the Negro River daily water level data. The resulting events are indicated by orange (droughts) and blue (floods) vertical ticks at the bottom of the panel. (**B**) Time-varying frequency of the identified droughts and floods between 1903 and 2015. (**C** and **D**) Duration, severity, and frequency of flood and drought events. The dotted lines denote the bivariate return period of a drought or flood event with a given duration or intensity. The dates of the events are indicated by the last two digits of the year of occurrence (for example, 12 for 2012). Events during the 21st century are indicated by red triangles.

To document and attribute the changes in hydrological drought and flood characteristics, we use continuous daily records of water levels from the Negro River at the historic port of Manaus from 1903 to 2015 and the Amazon River at Óbidos from 1970 to 2015 (fig. S1), as well as precipitation and atmospheric reanalysis data (see Materials and Methods). Long-term precipitation records are scarce in this vast tropical region, but these high-quality gauging records integrate precipitation and runoff across most of the basin and are the most suitable series for analysis of long-term changes in hydrological extreme events along the upper and lower sections of the Amazon River (*5*, *12*). Precipitation data during recent decades, with better station coverage, show a general tendency for wet seasons to get wetter and dry seasons to get drier, a pattern that is highly consistent with the observed increase in amplitude between peak and minimum flows of the Amazon River since the 1990s (fig. S2) (*2*). It is known that despite differences in catchment size and timing of the flood pulse, water levels at the Manaus station on the Negro River measure fluctuations of the Solimões-Amazon main stem (fig. S1) due to the so-called backwater effect (*12*, *13*). To verify this hydrological feature, we tested whether water levels on the Negro River at Manaus and water levels on the Amazon River downstream of Óbidos showed a similar pattern of variability during the period of overlap. As expected, variations in minimum and maximum seasonal water levels are highly correlated at the two gauging stations (*r* = 0.98 and 0.93, respectively; fig. S2). However, starting around 2006, the increase in maximum seasonal water levels has been slightly stronger at the downstream station of Óbidos because of the additional contribution of recent flooding in the Madeira River (*10*, *11*). The good agreement between the two gauge records enables us to use the long record of Manaus to quantify changes in hydrological extreme events in the Amazon River over the last century (Fig. 1A).

Mean daily water levels at the Manaus Port show a significant (*P* < 0.01) increasing trend of about 1 m (5% of the mean) during the 113 years of records (Fig. 1A). We used the critical river levels from the Geological Survey of Brazil (CPRM) to identify severe droughts (15.8 m) and floods (29.0 m) in Manaus and characterize their frequency, duration, and severity (see Materials and Methods). These river levels are critical for the functioning of the port and are used to declare emergency status in the city. We found 14 severe hydrological droughts and 14 severe floods since 1903 (Fig. 1A). A time-varying return period analysis (see Materials and Methods) indicates that the frequency of severe floods steadily increased since around 1970, leading to exceptionally high levels of flood hazard in recent years (Fig. 1B). We estimate that, over the study period, flood frequency has experienced a significant fivefold increase (*P* = 0.007, Cox-Lewis test for the null hypothesis of constant occurrence rate; see Materials and Methods), from roughly one flood every 20 years during the first half of the 20th century to one about every 4 years from the 2000s onward. In contrast, the frequency of severe hydrological droughts does not show any significant long-term change over the study period (*P* = 0.94, Cox-Lewis test), typically fluctuates between 5 and 12 years (Fig. 1B). Drought frequency was higher in the early 1900s, declined to a minimum in the wet decade of the 1970s, and then slightly increased again since the mid-1990s. These long-term changes have led to a doubling in frequency of extreme hydrological events since 2010 (gray in Fig. 1B), with a flood or drought now occurring nearly every other year. Except for the drought of 2010 and average hydrological conditions in 2011, there has been a flood every year from 2009 until the end of our study period in 2015 (Fig. 1, A to C), suggesting that the ongoing changes are highly anomalous. Varying the critical thresholds for defining floods and droughts results in different number of events but does not change the significance of the time-dependent trend in flood frequency; still, no temporal trend in drought frequency emerges over the past 50 to 100 years (fig. S3).

An examination of the characteristics of individual flood events indicates that recent floods not only occur more often but also have become more severe (Fig. 1, A to C). The exceptional floods of 2009 and 2012 (*4*) have no historical precedent in their duration and severity (Fig. 1C). A probabilistic copula analysis (see Materials and Methods) of severity and duration indicates that floods with these extreme characteristics should have, on average, a joint return period of about 50 to 60 years (that is, the return period of a flood event exceeding either a duration of 70 days or a water level of 29.7 m), yet they occurred within an interval of only 3 years. In contrast, the duration and severity of recent drought events (that is, post 2000) are not unusual considering the whole 1903–2015 period (Fig. 1D). The worst droughts on record, as seen solely from minimum seasonal water levels, occurred in 1906, 1963, and 2010 (*4*). The droughts of 1963 and 2010 had very similar severities (13.63 to 13.64 m) and durations (53 to 56 days) in Manaus, while that of 1906 was the longest (66 days). Droughts with these characteristics have also an average joint return period of between 50 and 60 years (Fig. 1D).

It is well known that extreme seasonal droughts in Amazonia are usually linked to either the El Niño events in the tropical Pacific or warm sea surface temperature (SST) anomalies in the tropical North Atlantic (*4*). We find that among several indices of Pacific and Atlantic climate variability, only the Atlantic Multidecadal Oscillation (AMO) (*14*) and the cross-equatorial Atlantic SST gradient (*15*) during the dry season are significantly and negatively correlated with variations in minimum seasonal water levels in Manaus, while moderate negative correlations with SSTs over the Niño 3.4 region occur during the preceding wet season (see fig. S4). The persistent negative correlations with Atlantic indices reflect the well-documented negative response of dry season precipitation over large regions of Amazonia and minimum Amazon River flows to warm SST anomalies in the tropical North Atlantic due to a northward displacement of the intertropical convergence zone (*4*, *16*). Decadal-scale changes in this cross-equatorial SST gradient and the AMO closely match the observed decadal fluctuation in drought frequency since the 1920s, with higher frequency of droughts during the periods of warmer tropical North Atlantic SSTs and positive phase of the AMO (fig. S5). This pattern is consistent with covariation between extremes in dry season precipitation over western Amazonia and the phase of the north-south gradient in tropical Atlantic SSTs (*17*–*19*), highlighting the dominant role of the tropical Atlantic in modulating decadal recurrence of hydrological droughts along the Amazon River.

**Fig. 2:**
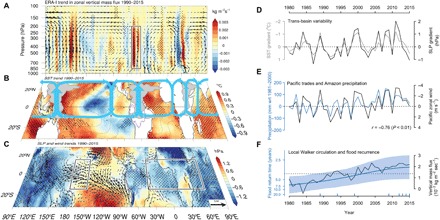
Intensification of the equatorial Walker circulation and increased flooding in Amazonia. (**A**) Trends in local Walker circulation (shading) (*25*) during the wet season (December to February) based on meridionally averaged (10°S–10°N) zonal vertical mass flux in the ERA-Interim (ERA-I) reanalysis. Overlying vectors represent the wet season climatological zonal wind and the vertical velocity scaled by a factor of −50. (**B**) Observed wet season SST trends from the ERSST (extended reconstructed SST) data set (*39*) and schematic representation of the overturning Walker cells in blue. (**C**) Trends in wet season SLP and wind stress from ERA-Interim reanalysis (*26*). (**D**) Tropical trans-basin variability indices [Pacific minus Atlantic SST and SLP anomalies averaged over the boxes in (C)] (*24*). (**E**) Comparison between September–February tropical Pacific trade winds over the region 5°S–5°N/160°E–180°E and wet season precipitation anomalies averaged over Amazonia. (**F**) Observed change in flood recurrence and strength of the ascending branch of the Walker circulation over Amazonia during the wet season, as represented by the mean zonal vertical mass flux averaged over the box in (A). All linear trends are given as the cumulative change over 26 years (1990–2015), and stippling indicates significance at the 90% confidence level.

In contrast to the mechanisms causing severe droughts, the mechanism for the observed change toward more severe floods is not well understood. It is known that historical maximum annual water levels in Manaus are related to SST variability in the Pacific (fig. S4) (*4*, *5*), reflecting remote atmospheric forcings of wet season rainfall in the basin (*16*). Since the early 1990s, tropical SST has shown a strong warming trend over the tropical Atlantic and Indo-western Pacific but La Niña–like cooling over the eastern Pacific (Fig. 2B) (*2*, *20*). These trends resulted in an increase in the interbasin contrast in SST and surface pressure (Fig. 2, C and D). Recent studies have mechanistically linked these interbasin trends to an ongoing intensification of the global atmospheric Walker circulation (*21*) and associated unprecedented strengthening of trade winds in the Pacific (Fig. 2, A and C) (*22*). The onset of the interbasin climate contrast and accelerated trade winds in the Pacific is consistent with the change in sign of the Interdecadal Pacific Oscillation (IPO) in the late 1990s (fig. S6) (*23*). However, the wind trends have been even stronger and larger scale than those typically associated with the IPO, with the IPO accounting for only about half the magnitude of the observed change (*22*). The rapid warming of the tropical Atlantic during this period has emerged as the key driver in initiating this major pantropical ocean-atmosphere reorganization (*20*) by intensifying the La Niña–type response in the tropical Pacific (Fig. 2, B and C).

Because the overturning Walker circulation connects the tropical ocean basins and has an ascending branch right over Amazonia, we expect a physical link between increased atmospheric overturning circulation and intensified deep convection and flooding in the region. We find that wet season (December to February) precipitation in Amazonia is strongly correlated with the intensity of the trade winds in the tropical Pacific (*r* = −0.76 with 10-m zonal wind in the region 5°S–5°N, 160°E–180°E; Fig. 2E), a surface expression of the Pacific Walker circulation cell. Variations in maximum annual water levels in Manaus are also consistently correlated with the Pacific trade winds and indices of Pacific/Atlantic sea level pressure (SLP) and SST gradients (*24*) connected with the Walker circulation (fig. S4).

To further examine this plausible mechanism, we computed the local Walker circulation (*25*) by decomposing the total vertical mass flux from the European Centre for Medium Range Weather Forecast (ECMWF) Interim (ERA-Interim) (*26*) reanalysis into orthogonal components associated with overturning in the meridional and zonal directions over the tropical band 10°S–10°N (see Materials and Methods). We find a significant linear trend in the rate of air mass upwelling in the ascending branches located over the central and western Amazon, eastern Africa, and the Maritime Continent since the early 1990s (Fig. 2A). Remarkably, the intensification of the Walker circulation averaged over Amazonia during the December to February wet season is highly consistent with our observed trend toward increased flooding in Manaus (Fig. 2F) and the associated increase in basin-wide wet season precipitation (fig. S2A). Similar analyses using the National Centers for Environmental Prediction–Department of Energy Atmospheric Model Intercomparison Project Reanalysis (NCEP-2) (*27*) and National Oceanic and Atmospheric Administration (NOAA) 20th century (*28*) reanalysis products agree very well and thus confirm the significance of the enhancement of upward motion in these regions (fig. S7). The onset of intensification of the Walker circulation also coincides with an observed 55% increase in wet day frequency over the rainiest parts of western Amazon since 1995 (*29*). Thus, we attribute the ongoing unprecedented increase in flooding in Amazonia to a strengthening of the Walker overturning circulation. This effect is centered over the western and central Amazon, and we expect it to dominate over drying trends in the southern margins of the basin, which are likely related to the marked change in land use over the past decades (*5*, *6*). Other factors, such as deforestation, hydropower dams, long-term changes in sediment loads and river cross sections near the gauges, and basin-wide changes in plant transpiration, might have also contributed to this flooding trend in flooding, but their relative importance should be evaluated using a suitable hydrological modeling approach.

## DISCUSSION

**Fig. 3:**
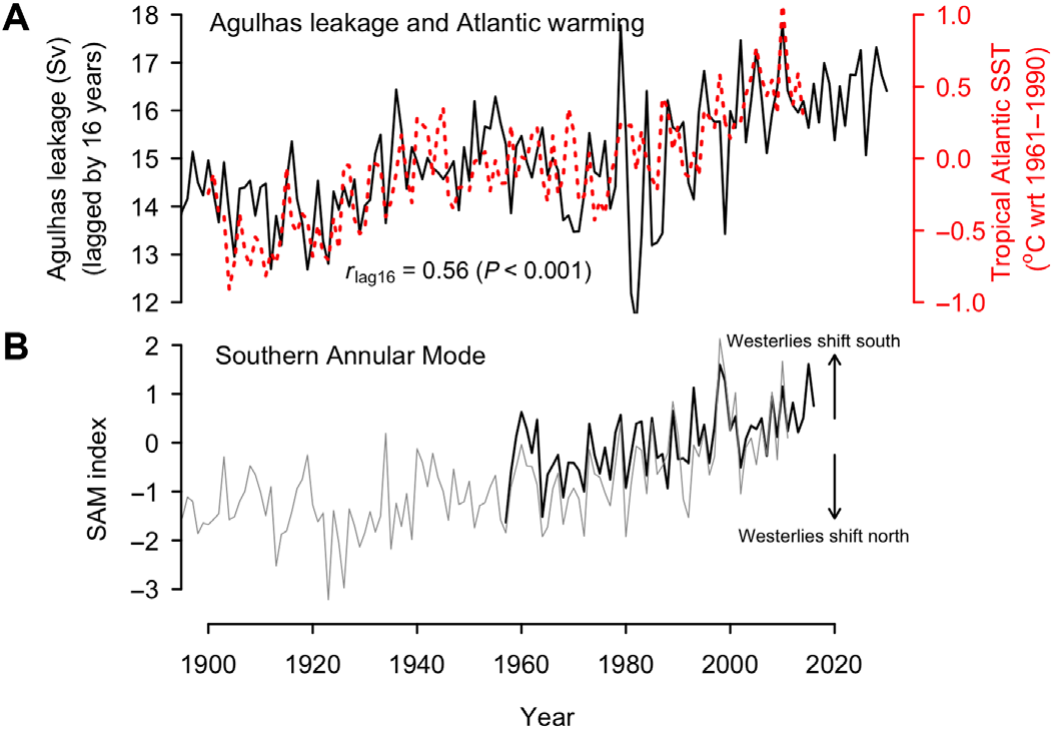
Atlantic warming and recent increase in Agulhas leakage with the progressive poleward displacement of the Southern Hemisphere westerlies. (**A**) Annual mean (January to December) SSTs in the tropical Atlantic (5°S–20°N) compared with annual Agulhas leakage (*34*) lagged by 16 years. (**B**) Annual mean Southern Annular Mode (SAM) from observations (black) (*40*) and NOAA 20th century reanalysis (gray) (*28*). wrt, with respect to.

Our statistical analyses suggest that the recent strengthening of the Walker circulation plays a key role in driving the observed increase in frequency and intensity of severe floods in the Amazon. This change in circulation is, in turn, part of a tropical-wide climate reorganization triggered most likely by rapid tropical Atlantic warming during recent decades (*20*). A combination of several factors has been invoked to explain the sustained warming of the tropical and north Atlantic sectors (*20*), including changes in radiative forcing (greenhouse gases increase combined with changes in aerosols) and natural variation in the AMO (fig. S6) linked to the Atlantic meridional overturning circulation. However, analysis of observations of the surface energy balance in the tropical Atlantic suggests that the ocean has actually lost heat to the atmosphere during the period of rapid warming (*30*), implying that oceanic circulation, instead of radiative forcing, must be the major contributor to the warming trend and the associated acceleration of the easterly winds over the region. In line with this inference, a possible, albeit underlooked, mechanism that might have contributed to Atlantic warming is the increased northward transport of warm Indian Ocean waters leaked into the Atlantic via the Agulhas Current around the tip of Africa (Fig. 3A) (*31*). Northward transport of these waters has been facilitated as a result of a southward shift of the westerly wind belt around Antarctica over recent decades, which has been attributed to the ozone hole and anthropogenic greenhouse warming (Fig. 3B) (*31*, *32*). The Agulhas leakage warming mechanism is also consistent with the results of hindcast experiments with artificially increased Agulhas leakage in a high-resolution ocean eddy-resolving climate model (*33*). A significant covariation of Agulhas leakage with tropical Atlantic SSTs on a lead time of 16 years (Fig. 3A) lends support to a possible contribution of this mechanism. A similar pattern of covariation with the AMO has been reported earlier at this time scale, which is physically consistent with transit time analyses of Agulhas leakage into the North Atlantic (*34*).

What are the implications of our results for future predictability of severe floods? Our analyses highlight the mechanistic connection between unprecedented flooding in Amazonia and the recent anomalous interbasin SST and SLP contrast likely driven by Atlantic warming (*20*, *21*, *24*). Unlike the limited predictability of ENSO (El Niño–Southern Oscillation) of about 1 year, the trans-basin climate variability can be predicted up to 3 years ahead in the current state-of-the-art climate prediction system (*24*). This feature can be exploited to estimate the probability of flooding extremes in Amazonia for several years ahead. On a longer time scale, the decadal cooling trend over the eastern tropical Pacific might currently be ending with the apparent switch to a warm phase of the IPO around 2014 (*35*). In contrast, the warming trend of the tropical Atlantic is likely to continue over the next few decades as a result of a combination of anthropogenic forcing, natural variability associated with the AMO, and the lagged response to the Agulhas leakage under continued poleward shift of the Southern Hemisphere westerly winds with anthropogenic forcing (*32*). Overall, on the time scale of a decade or so, we expect a weakening of the Pacific/Atlantic thermal contrast and thus a decrease in the intensity of the Walker circulation and in severe flooding in Amazonia, but likely not to pre-1990 flooding levels because of continued Atlantic warming.

## MATERIALS AND METHODS

### River and precipitation data

Long-term daily water level data for the Negro River at Manaus Harbor were obtained from the Brazilian Water Agency [Agência Nacional de Águas (ANA); www.snirh.gov.br/hidroweb/publico/medicoes_historicas_abas.jsf] for the period 1903–2015. Quality-controlled daily water levels for the Amazon River at Óbidos were obtained from the Environmental Research Observatory SO HYBAM (www.ore-hybam.org) for the period 1970–2015. Both records were screened for inconsistencies; other than a few typing errors detected in the record of Manaus, no quality problems were found. The Manaus gauge has been maintained at the same site by Manaus Harbor Ltd./Portobras since its installation in 1902. Changes in the river bed in the low-sediment Negro River have likely been small (*13*). The annual difference between high and low water levels may reach 15 m, and riverine life and navigation follow the pace of the predictable annual flood pulse. Disruptions to this cycle cause great impacts to riverine population, regional economy, and ecology (*4*, *9*, *11*). We chose to use the stage record directly rather than to derive discharge estimates (*13*) because water levels are less uncertain and extreme anomalies are more easily related to the impacts felt by the riverine populations. Discharge estimates become unrealistic when water overflows to the large floodplains during the wet season.

An average of dry and wet season precipitation for the Amazon basin was computed over the period 1970–2015 using the Brazilian network of rain gauges and four global gridded precipitation products (fig. S2). The network of rain gauges covers only the Brazilian Amazon and was created by combining data from the Brazilian Water Agency and the Brazilian National Institute of Meteorology (INMET; www.inmet.gov.br/). Only stations with a minimum of 30 years with complete data during the dry (*n* = 278) and wet (*n* = 215) seasons were retained. To avoid biasing the average toward regions with higher station coverage, the available stations for a given season and year were first averaged into 5° × 5° latitude-longitude boxes, and then the basin average for that season and year was computed by averaging all the grid boxes.

### Analysis of hydrological extreme events

Hydrological drought and flood extreme events were quantitatively defined to occur when daily water levels in Manaus fall below 15.8 m or rise above 29.0 m, respectively. These thresholds are used by the CPRM to monitor severe droughts and floods in this gauge and correspond to the long-term mean of minimum and maximum annual values ±1 SD. Furthermore, when water levels at Manaus reach 29 m, the municipality declares emergency status as the lower parts of the city start to be flooded. The duration and intensity of a drought (flood) event were determined as the number of days with water levels below (above) 15.8 m (29.0 m) and the minimum (maximum) daily water level reached during the event, respectively.

Temporal changes in the occurrence rates of drought and flood events were estimated using a nonparametric Gaussian kernel technique (*36*, *37*). Here, the estimated time-dependent occurrence rate, λ(*t*), corresponds to the probability of an extreme event per time interval, and thus its inverse gives the time-dependent return period. Kernel smoothing over a window of 15 years was used to explore decadal and longer-term changes in λ(*t*) together with their 90% confidence intervals from 2000 bootstrap simulations by resampling the list of yearly events with replacement. This technique is especially suited for modeling nonstationary processes such as our peak-over-threshold chronologies of extreme events (*37*). The Cox-Lewis statistic (*36*) was used to test the null hypothesis of constant occurrence rate over the observation interval (1903–2015) against the alternative hypothesis of a monotonic trend in the occurrence rate. The bivariate return periods of drought and flood events based on their observed duration and intensity were estimated using a two-dimensional Gumbel copula analysis (*38*).

### Tropical climate variability and atmospheric circulation

Trends in tropical SST from 1990 to 2015 (Fig. 2A) were estimated using the extended reconstructed SST version 3b data set (*39*). The influence of large-scale climate forcings on hydrological variability in the basin was quantified using monthly correlations of maximum and minimum seasonal water levels with Atlantic and Pacific climate indices (fig. S4). The ERA-Interim reanalysis (*26*) data were used to estimate trends in tropical SLP, surface (10 m) winds, and atmospheric circulation (Fig. 2, B, C, E, and F).

The overturning atmospheric circulation over the tropical band 10°S–10°N was decomposed into the local Walker and Hadley circulations (*25*). This approach separates the vertical velocity, or equivalently mass flux, field into independent components in meridional and zonal directions. To verify the robustness of the circulation changes identified in the ERA-Interim reanalysis, the local Walker circulation was also computed for the NCEP-2 (*27*) and NOAA 20th century (*28*) reanalyses. Here, the continuity equation is expressed in pressure coordinates

$$(\frac{\partial \mu }{\partial x}+\frac{\partial \upsilon }{\partial y})p+\frac{\partial \omega }{\partial p}=0$$ (1)

where *p* the is pressure, *x* and *y* are the Cartesian coordinates, $\omega \equiv \frac{dp}{dt}$ is the analog/generalization of vertical velocity for pressure coordinates, and partial derivatives with respect to *x* and *y* are taken along the surfaces of constant pressure. The vertical branch of the overturning circulation is the divergent (irrotational) component of the velocity field on constant pressure surfaces. According to a theorem by Helmholtz, a velocity field (μ,υ) can be decomposed into divergent and rotational components with divergent component (μ, υ)div = ∇χ, where χ is called the velocity potential. If we assume that $\chi =\frac{\partial \mu }{\partial p}$, then Eq. 1 becomes a Poisson’s differential equation for the potential μ with source term −ω

$$\nabla^{2}\mu_{p}=-\omega$$ (2)

This equation can be solved numerically for μ on fixed pressure surfaces given the observed vertical velocity ω. Defining further ψ ≡ − ∇_*p*_μ, Eq. 2 can be written as

$$\omega =-\nabla^{2}\mu_{p}=\nabla_{p}{\left(\psi \right)}_{x}\psi_{y}$$ (3)

which suggests the following partitioning of the vertical velocity ω into Walker and Hadley circulation components: $\omega_{x}=\frac{\partial \psi_{x}}{\partial x}=\frac{\partial^{2}\mu }{\partial x^{2}},\omega_{y}=\frac{\partial \psi_{y}}{\partial y}=\frac{\partial^{2}\mu }{\partial y^{2}}.$ This can be generalized to spherical coordinates to obtain

$$\omega_{\lambda }\;\text{sin}\;\vartheta =\frac{1}{a}\frac{\partial \psi_{\lambda }}{\partial \lambda }=\frac{1}{a^{2}\text{sin}\;\vartheta }\frac{\partial^{2}\mu }{\partial \lambda^{2}}$$ (4)

$$\omega_{\vartheta }\text{sin}\;\vartheta =\frac{1}{a}\frac{\partial (\psi_{\vartheta }\text{sin}\;\vartheta )}{\partial \vartheta }=\frac{1}{a^{2}}\frac{\partial }{\partial \vartheta }(\text{sin}\;\vartheta \frac{\partial \mu }{\partial \vartheta })$$ (5)

where λ and *ϑ* are azimuth angle and polar angle (angle to *z* axis), respectively, *a* is Earth’s radius, and ω_λ_ and ω_ϑ_ are the vertical velocity components in meridional and zonal planes, respectively.

## Supplementary Material

http://advances.sciencemag.org/cgi/content/full/4/9/eaat8785/DC1

## SUPPLEMENTARY MATERIALS

Supplementary material for this article is available at http://advances.sciencemag.org/cgi/content/full/4/9/eaat8785/DC1 Fig. S1. Location of Manaus and Óbidos gauges in the Amazon system. Fig. S2. Increasing seasonal hydrological variability in Amazonia during 1970–2015. Fig. S3. Time-varying frequency of floods (left) and droughts (right) between 1903 and 2015 using different thresholds to define extreme events. Fig. S4. Correlations of monthly Pacific and Atlantic climate indices with seasonal water levels of the Negro River at Manaus and the Amazon River at Óbidos. Fig. S5. Decadal fluctuations in drought frequency in Amazonia and Atlantic climate modes. Fig. S6. Comparison of tropical Atlantic and Pacific SST averages along with the IPO and AMO indices during the Amazon wet season. Fig. S7. Trends and average time series of local Walker circulation based on meridionally averaged (10°S–10°N) zonal vertical mass flux in the ERA-Interim (ERA-I), NOAA 20th century (N20CR), and NCEP-2 (NCEP) reanalyses. References (*41*, *42*)
