# Supplementary material for: Recent intensification of Amazon flooding extremes driven by strengthened Walker circulation
Source: Sci Adv. 2018 Sep 19;4(9):eaat8785. doi: 10.1126/sciadv.aat8785 (PMC6155052; doi:10.1126/sciadv.aat8785)
Supplement: http://advances.sciencemag.org/cgi/content/full/4/9/eaat8785/DC1 [file supp_4_9_eaat8785__index.html]

Science Advances | Science Advances

## Supplementary Materials

**This PDF file includes:**

- Fig. S1. Location of Manaus and Óbidos gauges in the Amazon system.
- Fig. S2. Increasing seasonal hydrological variability in Amazonia during 1970–2015.
- Fig. S3. Time-varying frequency of floods (left) and droughts (right) between 1903 and 2015 using different thresholds to define extreme events.
- Fig. S4. Correlations of monthly Pacific and Atlantic climate indices with seasonal water levels of the Negro River at Manaus and the Amazon River at Óbidos.
- Fig. S5. Decadal fluctuations in drought frequency in Amazonia and Atlantic climate modes.
- Fig. S6. Comparison of tropical Atlantic and Pacific SST averages along with the IPO and AMO indices during the Amazon wet season.
- Fig. S7. Trends and average time series of local Walker circulation based on meridionally averaged (10°S–10°N) zonal vertical mass flux in the ERA-Interim (ERA-I), NOAA 20th century (N20CR), and NCEP-2 (NCEP) reanalyses.
- References (*41*, *42*)

Download PDF

**Files in this Data Supplement:**

- Adobe PDF - aat8785\_SM.pdf
