# Supplementary material for: Recent intensification of Amazon flooding extremes driven by strengthened Walker circulation
Source: Sci Adv. 2018 Sep 19;4(9):eaat8785. doi: 10.1126/sciadv.aat8785 (PMC6155052; doi:10.1126/sciadv.aat8785)
Supplement: http://advances.sciencemag.org/cgi/content/full/4/9/eaat8785/DC1 [file aat8785_SM.pdf]

Science Advances
AAAS

advances.sciencemag.org/cgi/content/full/4/9/eaat8785/DC1

Supplementary Materials for

Recent intensification of Amazon flooding extremes driven by strengthened Walker circulation

Jonathan Barichivich*, Emanuel Gloor, Philippe Peylin, Roel J. W. Brienen, Jochen Schöngart, Jhan Carlo Espinoza, Kanhu C. Pattnayak

*Corresponding author. Email: campsidium@gmail.com

Published 19 September 2018, *Sci. Adv.* **4**, eaat8785 (2018)
DOI: 10.1126/sciadv.aat8785

This PDF file includes:

Fig. S1. Location of Manaus and Óbidos gauges in the Amazon system.
Fig. S2. Increasing seasonal hydrological variability in Amazonia during 1970–2015.
Fig. S3. Time-varying frequency of floods (left) and droughts (right) between 1903 and 2015 using different thresholds to define extreme events.
Fig. S4. Correlations of monthly Pacific and Atlantic climate indices with seasonal water levels of the Negro River at Manaus and the Amazon River at Óbidos.
Fig. S5. Decadal fluctuations in drought frequency in Amazonia and Atlantic climate modes.
Fig. S6. Comparison of tropical Atlantic and Pacific SST averages along with the IPO and AMO indices during the Amazon wet season.
Fig. S7. Trends and average time series of local Walker circulation based on meridionally averaged (10ºS–10ºN) zonal vertical mass flux in the ERA-Interim (ERA-I), NOAA 20th century (N20CR), and NCEP-2 (NCEP) reanalyses.
References (*41*, *42*)

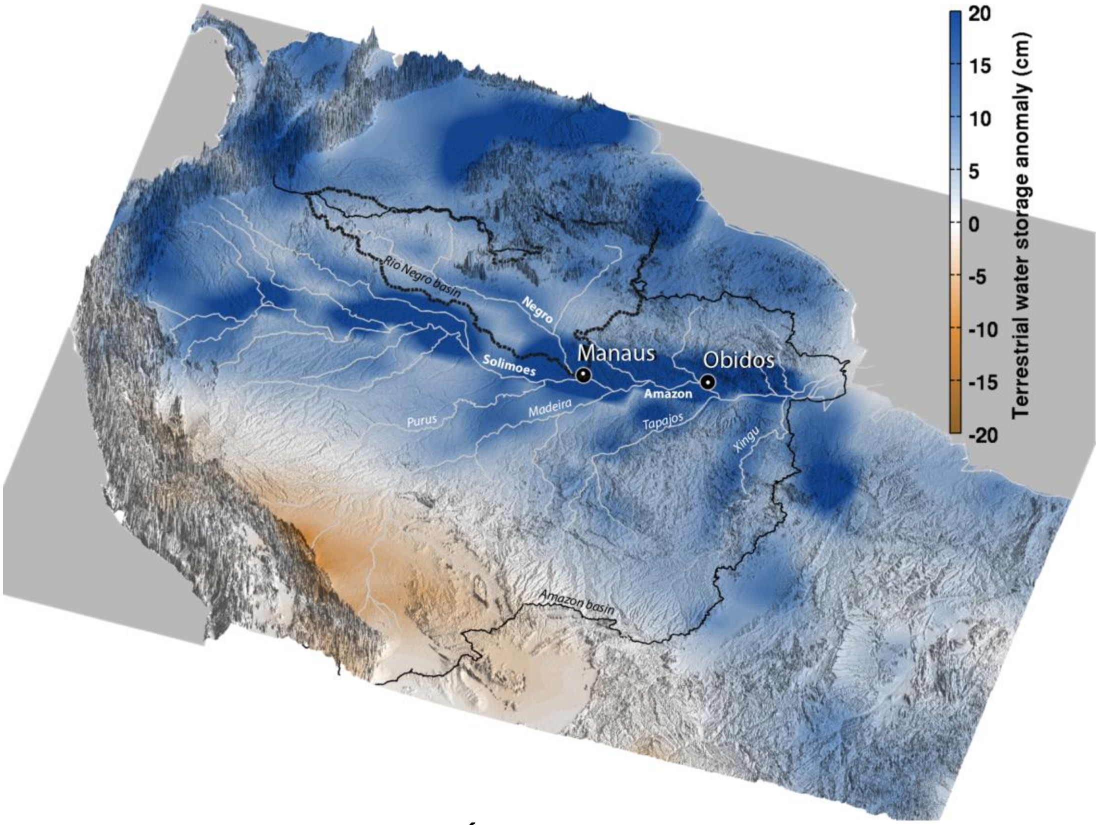

Fig. S1. Location of Manaus and Óbidos gauges in the Amazon system. The shading represents the Jan-Mar anomaly in terrestrial water storage from GRACE satellites (*41*) during the extreme flooding event in 2012. Note the strong positive anomaly (blue) along the floodplains of the main stem of the upper (Solimões) and lower Amazon River.

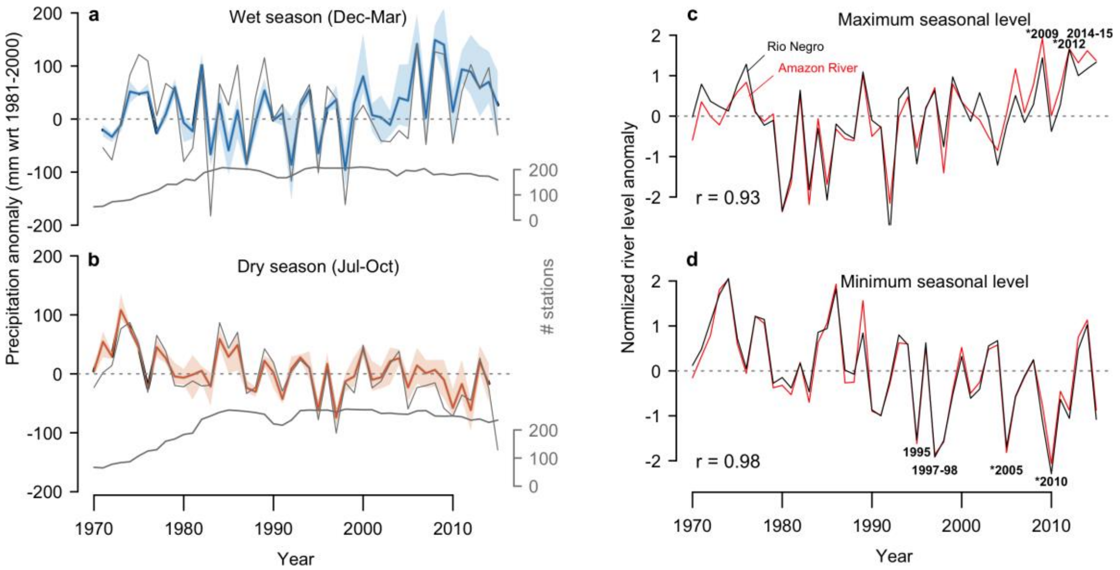

Fig. S2. Increasing seasonal hydrological variability in Amazonia during 1970–2015. (a and b) Mean wet and dry season precipitation variability in the basin based on in-situ rain gauges (gray) and the average of gridded estimates (colour). Shading indicates the range of variation of four gridded precipitation datasets (UDel v4.01, GPCC v7, CRU TS 3.24, ERA-Interim). (c and d) Maximum and minimum annual water levels of Negro river at Manaus (black) and the Amazon River at Óbidos (red). Major flood and drought years are indicated in each panel. The asterisk denotes the so-called 'once in a century' (4) events.

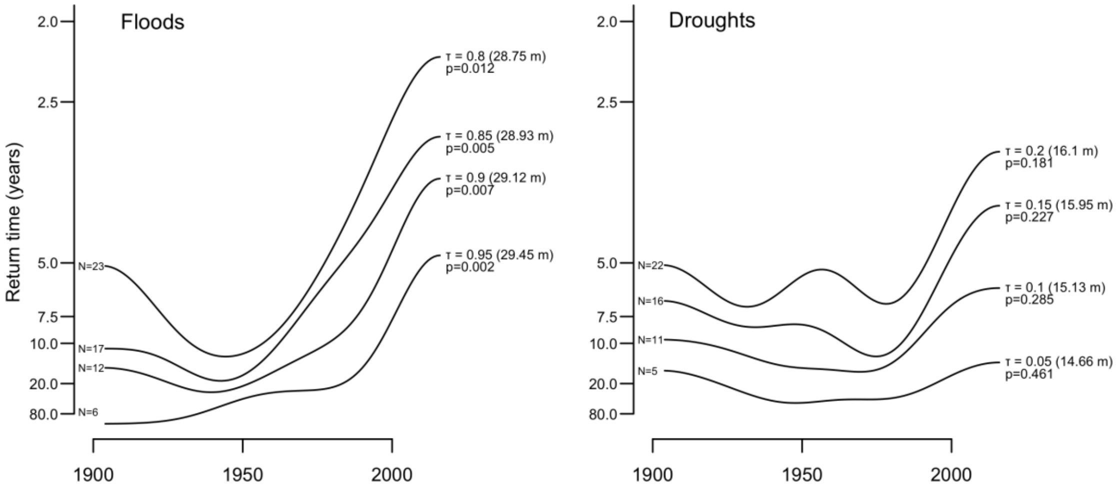

Fig. S3. Time-varying frequency of floods (left) and droughts (right) between 1903 and 2015 using different thresholds to define extreme events. The thresholds correspond to the lower and upper 5th, 10th, 15th and 20th quantile (τ) of daily water levels. The corresponding water level is given in brackets and the resulting number of events is shown in the left side of each panel. The p-value of the Cox-Lewis test for the null hypothesis of constant occurrence rate is also shown for each case. Note that the time-dependent trend in flood frequency is highly significant in all cases whilst there is no case with a significant trend in drought frequency for the study period as a whole.

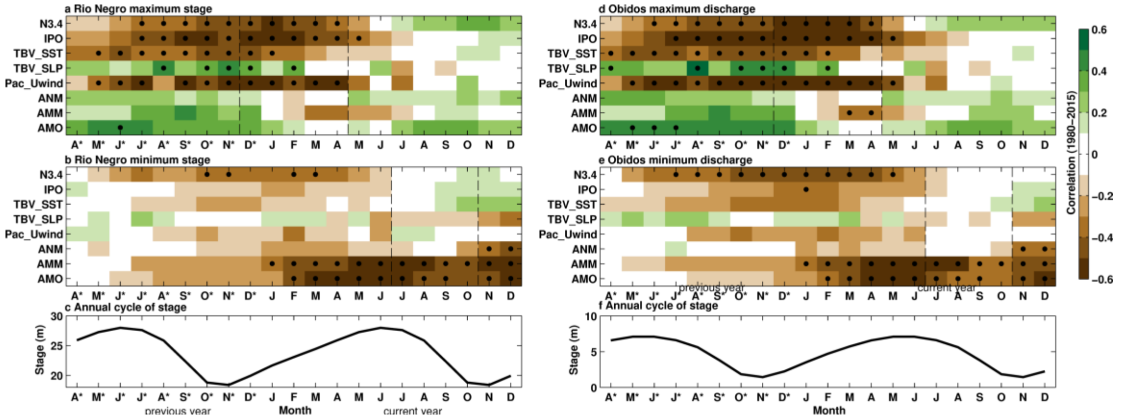

Fig. S4. Correlations of monthly Pacific and Atlantic climate indices with seasonal water levels of the Negro River at Manaus and the Amazon River at Óbidos. (a and d) Correlations for maximum seasonal water levels. (b and e) Correlations for minimum seasonal water levels. (c and f) Seasonal cycle of water levels. Correlations are shown from April of the previous calendar year to December of the current year. The *N3.4* (*42*) and the *Inter-decadal Pacific Oscillation index (IPO)* (*23*) represent tropical and Pacific-wide Sea Surface Temperature variability, respectively. The tropical Trans-basin Variability indices (*21*, *24*) on the basis of SLP *(TBV SLP)* and SST *(TBV SST)* are defined as the zonal gradient of SLP and SST anomalies averaged in each region between the tropical central Pacific (15°S–15°N, 180°W–150°W) and the tropical Atlantic-Indian Ocean (15°S–15°N, 40°W–60°E). *Pac Uwind* represents the strength of the Pacific trade winds and is the average of the 10-m zonal wind from the ERA-Interim reanalysis in the region 5°S-5°N, 150°–180°W (*22*, *24*). *Atlantic Niño Mode (ANM)* (*15*) represents an El Niño-like warming and cooling mode of variability in the tropical Atlantic. *The Atlantic Meridional Mode (AMM)* (*15*) measures the SST gradient between the North and South tropical Atlantic. *The Atlantic Multi-decadal Oscillation (AMO)* (*14*) is an oscillatory mode of SST variability in the North Atlantic with a 65–80 year cycle.

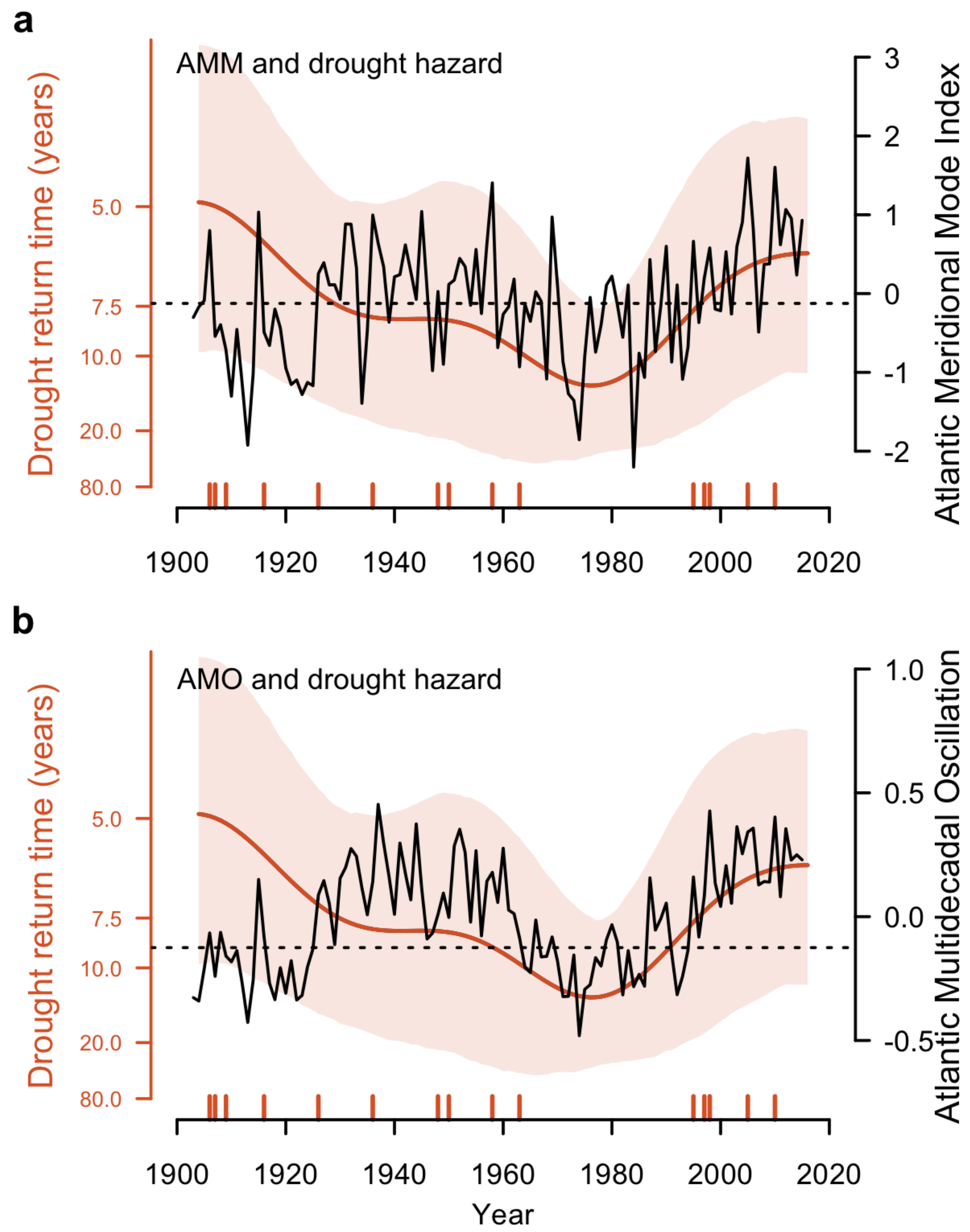

Fig. S5. Decadal fluctuations in drought frequency in Amazonia and Atlantic climate modes. (a) Observed changes in drought frequency and dry season (Jul-Nov) variability of the Atlantic Meridional Mode (*15*). (b) Observed changes in drought frequency and dry season Atlantic Multidecadal Oscillation.

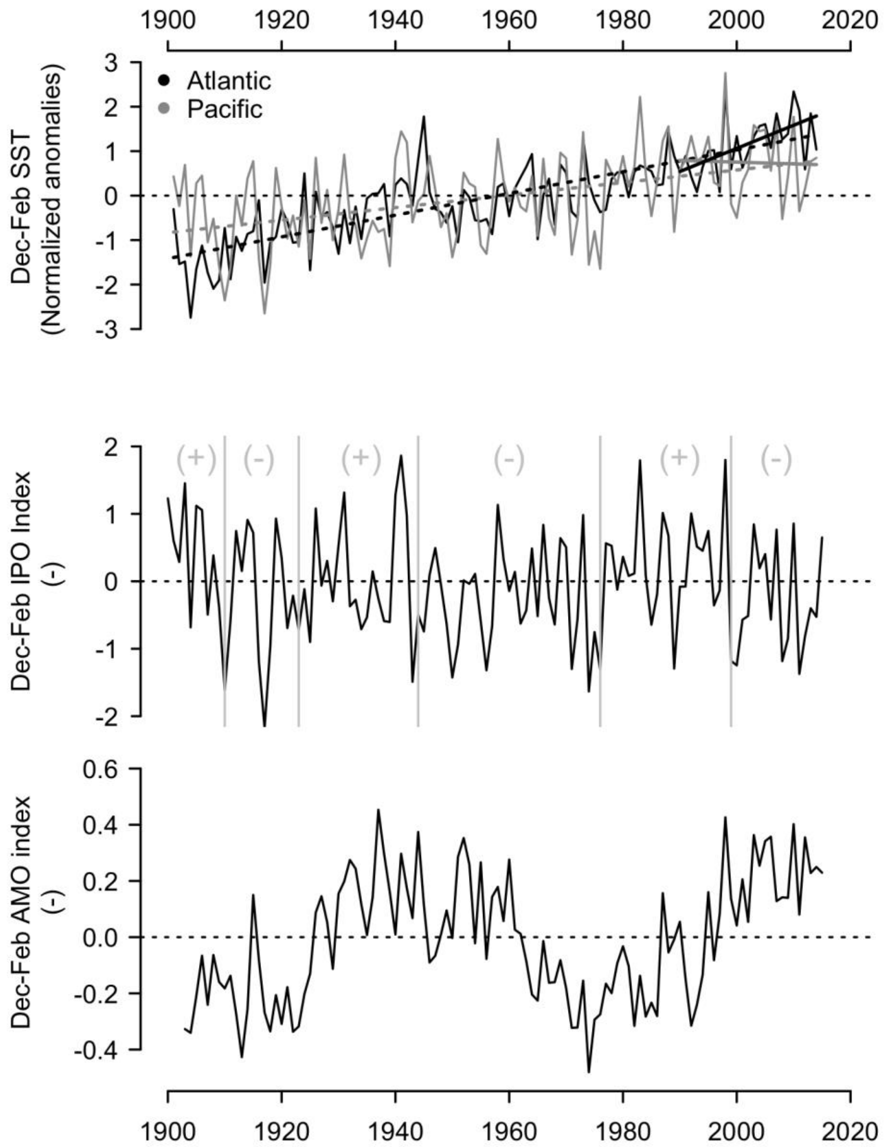

Fig. S6. Comparison of tropical Atlantic and Pacific SST averages along with the IPO and AMO indices during the Amazon wet season. The vertical gray lines and signs denote the phases of the IPO (*23*). The linear trends over the periods 1900-2015 (dashed) and 1990-2015 (bold) are shown. The spatial domains for the averages are 15°S–15°N/40°W–60°E for the Atlantic and 15°S–15°N/120°E–75°W for the Pacific.

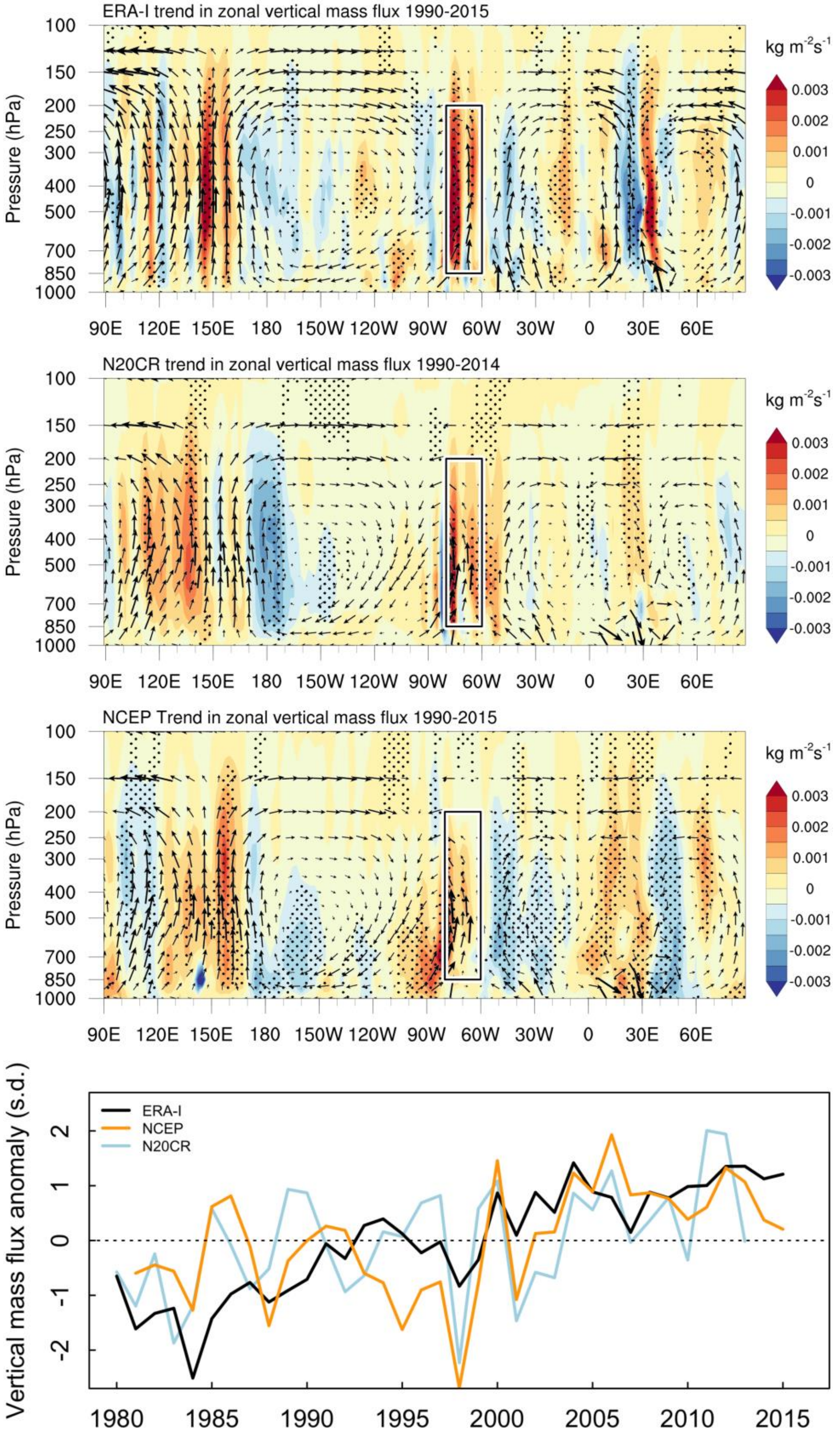

Fig. S7. Trends (shading) and average time series of local Walker circulation based on meridionally averaged (10°S–10°N) zonal vertical mass flux in the ERA-Interim (ERA-I), NOAA 20th century (*28*) (N20CR), and NCEP-2 (*27*) (NCEP) reanalyses. Overlying vectors represent the wet season climatological zonal wind and the vertical velocity scaled by a factor of -50. All linear trends are given as the cumulative change over 26 years (1990–2015) and stippling indicates significance at the 90% confidence level.
